# Digital Training for Mental Health Promotion in Young People With Climate Change-Related Distress: Protocol for a Feasibility Randomized Controlled Trial

**DOI:** 10.2196/77764

**Published:** 2025-12-05

**Authors:** Leonie Fleck, Henrik Wasmus, Frederike Schirmbeck, Claudia de Freitas, Raquel Teixeira, Liuska Sanna, Anne Marijn de Graaff, Manuela De Allegri, Hoa Thi Nguyen, Jan R Boehnke, Evaldas Kazlauskas, Wietse Tol, Ulrich Reininghaus

**Affiliations:** 1 Department of Public Mental Health Central Institute of Mental Health, Medical Faculty Mannheim Heidelberg University Mannheim Germany; 2 Center for Psychology at University of Porto (CPUP) University of Porto Porto Portugal; 3 Faculty of Psychology and Education Sciences University of Porto Porto Portugal; 4 EPIUnit – Institute of Public Health (ISPUP) University of Porto Porto Portugal; 5 Mental Health Europe Brussels Belgium; 6 Department of Mental Health, Brain Health and Substance Use World Health Organization Geneva Switzerland; 7 Heidelberg Institute of Global Health, University Hospital and Faculty of Medicine, University of Heidelberg Heidelberg Germany; 8 School of Health Sciences University of Dundee Dundee United Kingdom; 9 Faculty of Philosophy, Institute of Psychology Vilnius University Vilnius Lithuania; 10 Department of Public Health University of Copenhagen Copenhagen Denmark; 11 Health Service and Population Research Department Institute of Psychiatry, Psychology & Neuroscience King’s College London London United Kingdom; 12 ESRC Centre for Society and Mental Health King’s College London London United Kingdom; 13 German Center for Mental Health (DZPG), partner site Mannheim-Heidelberg-Ulm Mannheim Germany

**Keywords:** climate change, climate change distress, ecological momentary intervention, ecological momentary assessment, mental health promotion, mHealth, youth, feasibility, randomized controlled trial, mobile phone

## Abstract

**Background:**

Efforts in mental health research have long focused on the care and long-term outcomes of mental disorders. More recently, a shift in focus has occurred toward mental health promotion and prevention. One priority target population for promotion and prevention is youth with climate change–related distress. In light of the real-world threat of climate change, adaptive emotion regulation and engagement in meaningful action are 2 important strategies for promoting mental health. Ecological momentary interventions (EMIs) allow for the delivery of accessible interventions for young people with climate change–related distress, but evidence on their feasibility or beneficial effects is currently lacking.

**Objective:**

We aimed to examine the feasibility and initial signals of efficacy of the Climate Mind and Act (CliMACT) training, a novel hybrid EMI for mental health promotion in youth with climate change–related distress.

**Methods:**

A 2-arm, parallel-group, and assessor- and analyst-blinded feasibility randomized controlled trial (RCT) will be conducted in 50 young people aged 14-25 years with climate change–related distress, who will be allocated on a 1:1 ratio to the experimental condition (CliMACT training + care as usual [CAU]) or the control condition (CAU only). CliMACT involves 3 sessions with a mental health professional and 6-week access to a smartphone-based EMI to support the real-world transfer of training content based on compassion-focused interventions and acceptance and commitment therapy. The EMI delivery schemes involve enhancing (introducing new EMI components), consolidating (training of EMI components), and adaptive (triggered in moments of higher negative affect) components. CAU involves access to all standard health care and social services. Feasibility criteria of the trial methodology include recruitment, randomization, and retention. Feasibility outcomes of delivering the CliMACT training include participant satisfaction, participant adherence, and mental health professionals’ fidelity to the training protocol. Initial signals of efficacy on mental health candidate outcomes and mechanisms will be explored. As feasibility criteria for a priori planned subgroup analyses, credibility criteria will be established and distributions of indicators for health inequities explored. Feasibility criteria for measuring costs of care and service use and health-related quality of life for an economic evaluation in a future definitive RCT will include exploring response distributions across groups. Candidate outcomes and mechanisms will be assessed at baseline, post training, and 4-week follow-up, using self-report and 6 days of ecological momentary assessment.

**Results:**

The first enrollment took place in December 2024. Data collection was completed by August 25, 2025. Results are expected for publication in 2026.

**Conclusions:**

To our knowledge, this is the first study to establish the feasibility and initial signals of efficacy of an EMI, targeted specifically at young people with climate change–related distress. If feasibility can be established, the trial will inform a future fully powered efficacy-effectiveness RCT, accompanied by an economic evaluation.

**Trial Registration:**

ISRCTN ISRCTN33613914; https://doi.org/10.1186/ISRCTN33613914

**International Registered Report Identifier (IRRID):**

DERR1-10.2196/77764

## Introduction

### Background

Climate change is one of the current societal challenges recognized to pose a threat to mental health. While it is commonly agreed that negative emotional reactions toward the real threat of climate change are natural and common [1], in a substantial proportion of individuals, climate change–related distress is related to impairment in daily life, including difficulties with sleeping, concentrating, or fulfilling daily roles [2-5]. For example, in a large survey among young people in Germany, 65% reported negative emotions in relation to climate change, and about 20% reported experiencing sleep problems and a limited sense of joy due to their concerns about climate change [2]. These findings indicate that youth with climate change–related distress may be one priority target population, but scalable strategies for mental health promotion and prevention remain very limited.

The recent rapid advances in digital technologies offer new avenues for ecological translation (ie, daily life application) of accessible and scalable mental health promotion [6,7], including for young people with climate change–related distress. Ecological momentary interventions (EMI) provide a unique opportunity for real-life and real-world transfer of therapeutic, preventive, or promotional principles, tailored to the person, moment, and context [8-12]. Due to their accessible and personalized nature, EMIs are a promising intervention method for ecological translation to the living environments of vulnerable groups. Thus, EMIs are a potential scalable digital intervention option for young people with climate change–related distress. While overall, there is evidence on acceptability, safety, and efficacy of EMIs for the prevention and early intervention in young people with early mental health problems [13-16], this remains to be established among young people with climate change–related distress in a target group-specific and vulnerability-sensitive approach.

While there is a lack of evidence with interventions among the target group, conceptual work on fostering resilience in young people with climate change–related distress indicates that interventions focused on the individual should consist of 2 strands: one enabling adaptation, which includes the successful regulation of stressful emotions, and one focusing on action, enabling youth to engage in meaningful transformation of their environments and living conditions [17-19]. In a recent feasibility randomized controlled trial (RCT), we found strong evidence for the safety and feasibility of trial methodology and intervention delivery for EMIcompass, a novel, transdiagnostic EMI intervention based on principles of compassion-focused interventions (CFI) for enhancing emotional resilience in youth with early mental health problems [16]. The feasibility RCT also revealed initial signals of efficacy for reduced momentary stress reactivity, and improved quality of life and momentary emotional resilience. These findings support using and adapting training components from EMIcompass for targeting emotion regulation in the target population of young people with climate change–related distress. Evidence has further shown that focusing on action and, more specifically, on meaning-focused coping in relation to climate change is related to more positive mental health outcomes [20]. This could support incorporating principles of acceptance and commitment therapy (ACT) into a training geared toward promoting mental health in youth with climate change–related distress. This includes encouraging distressed youth to choose and engage in actions that are aligned with their own values, as proposed by Niessen and Peter [18], and is further supported by ecological momentary assessment (EMA) research showing that value-based action is associated with better well-being [21]. Interventions based on ACT have shown promising results as a transdiagnostic approach for reducing stress in young people [22]. There is further evidence to suggest that digital interventions based on ACT principles for young people have stronger effects in targeted as compared to universal strategies for intervention [23]. Therefore, the current study adopts a targeted approach to bolster the mental health of young people who experience climate change–related distress. Taken together, CFI and ACT offer promising intervention principles and techniques amenable to being implemented in an EMI for reducing climate change–related distress in young people, but evidence on the feasibility of its delivery in this target population is lacking.

As underlying candidate mechanisms of change, transdiagnostic third-wave behavioral interventions such as CFI and ACT use techniques expected to foster mental health by enhancing self-compassion, mindfulness, psychological flexibility, and emotion regulation [24-26]. As a further candidate mechanism, in this particular target group and proposed intervention, value-based action may manifest in proenvironmental behavior (PEB) [27]. Acquiring both CFI- or ACT-based skills and strategies may moreover promote rather nonspecific candidate mechanisms such as emotional resilience and self-efficacy. As candidate outcomes, reduction of psychological distress or symptoms is often considered a secondary rather than primary aim of third-wave behavioral interventions [24,28]. In line with extensive literature that mental health is much more than the absence of psychopathology [29], candidate outcomes of a mental health promotion or prevention training for young people with climate change–related distress ought to include indicators of positive mental health, such as mental well-being, quality of life (including the emotional and social domain), and happiness. In a second vein, candidate outcomes include climate change distress and impairment itself, as well as general psychological distress often associated with the former [30].

Building on a marginalized population approach, population-based strategies for improving mental health may inadvertently augment the long-documented inequities in health among marginalized groups, who, for example, due to social position, limited resources, or problems with access, may benefit less from such strategies [31,32]. Developing novel intervention and training approaches, therefore, requires us to monitor and mitigate the potential of exacerbation of inequities, which may be caused by differential reach of and effectiveness for groups in marginalized or vulnerable positions. Urbanicity, low socioeconomic status (SES), lower education, female gender, and migrant and ethnic minority group status reflect indicators of socioenvironmental and ethnic mental health inequities also highlighted in the PROGRESS (Place of Residence; Race, Ethnicity, Culture, or Language; Occupation; Gender or Sex; Religion; Education; Socioeconomic Status; and Social Capital)–plus framework for equity in health [33,34]. Crucially, they have also been reported to play a direct or indirect role in accelerating the impact of climate change on mental health [5,35-37]. Despite evidence on initial signals of efficacy for the EMIcompass intervention [16], evidence in young people with climate change–related distress on target group- and training-specific candidate outcomes and mechanisms, taking relevant indicators of inequities in health into account, is pending.

### This Study

This study aims to investigate the feasibility and initial signals of efficacy of the CliMACT (Climate Mind and Act) training, a novel hybrid EMI for mental health promotion in youth with climate change–related distress, in addition to access to all standard health care and social services (care as usual [CAU]), compared with CAU only in a feasibility RCT (ISRCTN33613914). Feasibility criteria are based on the principles of the CONSORT (Consolidated Standards of Reporting Trials) extension for pilot and feasibility trials [38] and adapted from the study by Reininghaus et al [16]. To achieve the overall aim, we will first, examine the feasibility of the trial methodology (based on recruitment, randomization, and retention). Second, we will examine the feasibility of delivering the CliMACT training (based on participant satisfaction, participant adherence, and fidelity to the training protocol) and explore its specific and nonspecific active ingredients (CliMACT training components; working alliance toward mental health professionals and toward the EMI). Third, we will explore signals of efficacy of the CliMACT training on candidate outcomes, that is, positive mental health (quality of life [psychological and social domain], happiness, and mental well-being), climate change distress and psychological distress, as well as on momentary affect and momentary climate change–related distress at posttraining and 4-week follow-up. Fourth, we explore signals of efficacy of the CliMACT training on candidate mechanisms of change, namely self-compassion, mindfulness, psychological inflexibility, self-efficacy, resilience, PEB, emotion regulation, internalized stigma, and experience of discrimination, self-stigma of help-seeking, as well as on momentary emotional resilience, momentary self-efficacy, momentary PEB, and momentary value-based living at posttraining and 4-week follow-up. Fifth, we examine the feasibility of establishing credibility criteria for an a priori planned subgroup analyses on social and ethnic inequities in health by exploring the distribution of indicators for health inequities relevant to climate change–related distress, and exploring the effects of the CliMACT training stratified by marginalized or vulnerable group status (defined as meeting any of the following criteria: gender not male, ethnic minority, migration history, low family SES, current urbanicity, or lower education level). Sixth, we examine the feasibility of measuring related costs of care and service use and health-related quality of life for the accompanying economic evaluation in a future definitive RCT, and explore the distribution of cost patterns across the experimental and control groups.

## Methods

### Cocreation

At the outset of this study, a society advisory group (SAG) including representatives from national mental health–related umbrella organizations, lived experience experts, policy makers, and mental health practitioners was set up in Mannheim, Germany. The SAG leads the research cocreation process in collaboration with the research team throughout the project. Throughout the project, the SAG will meet with the research team and be involved in decisions about this study’s design, interpretation of findings, knowledge integration, dissemination, and development of a scaling strategy. Consultation meetings will be held at least annually and at key decision points (eg, after completing analyses on feasibility and initial efficacy signals). Representatives from the group with lived experience will receive financial compensation for contributing their personal time. Before the start of the feasibility RCT, a set of potential scenarios for intervention was cocreated with SAG members based on desk reviews and consultation meetings. The intervention scenarios specified different target groups, recruitment sites, and strategies, and the training delivery mode. The selection of a final intervention scenario was carried out by SAGs and other stakeholders in the mental health field using a set of methods (ie, a Delphi panel and scenario-based workshops) that are described in detail elsewhere [39]. The scenario selected will target a broader group of young people with climate change–related distress, with recruitment strategies focusing on Mannheim and the Rhine-Neckar region, including sites as outlined in the Recruitment section. Stakeholders favored a hybrid intervention, including access to a smartphone-based EMI combined with facilitating face-to-face sessions with a mental health professional.

### Study Design

**Figure 1:**
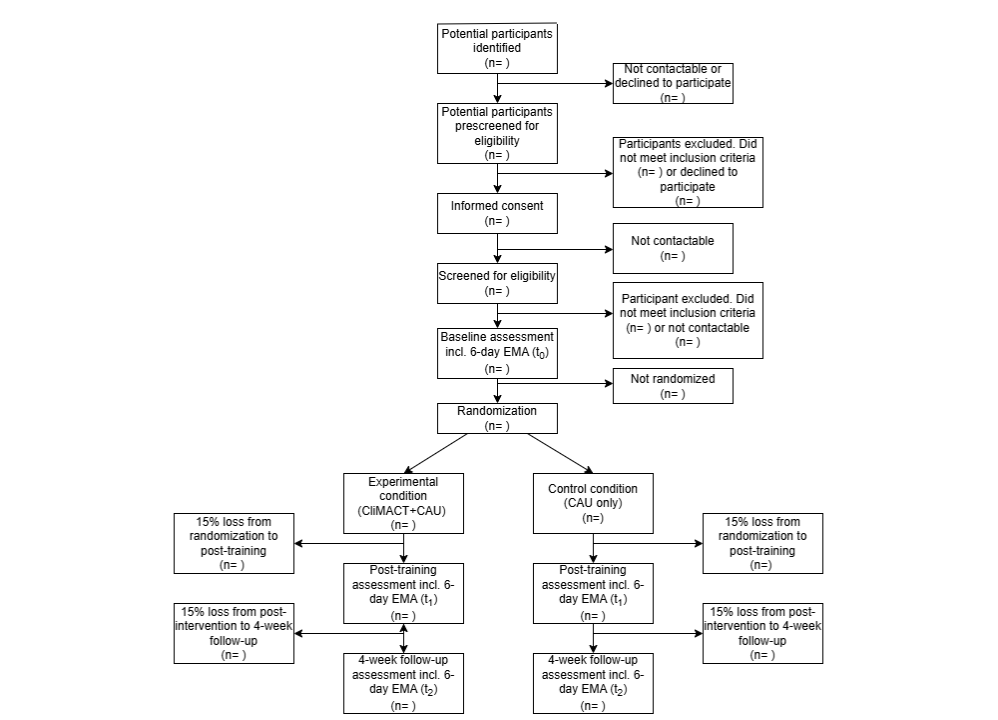
Study flowchart. CAU: care as usual; CliMACT+CAU: Climate - Mind and Act+Care as Usual; EMA: ecological momentary assessment; incl: including.

This feasibility study protocol follows the CONSORT 2010 statement extension to randomized pilot and feasibility trials (Multimedia Appendix 1). In a 2-arm, parallel-group, assessor- and analyst-blinded feasibility RCT, 50 individuals aged 14-25 years with climate change–related distress will be randomly allocated to (1) the CliMACT training in addition to CAU or (2) CAU only at a 1:1 ratio. Potentially eligible participants will be prescreened for eligibility in a phone call. Participants will then complete informed consent procedures with an authorized member of this study’s team, followed by the completion of questionnaires to establish eligibility. Next, outcome data will be collected at baseline (*t*_0_), at the end of the 6-week training period (*t*_1_), and at a 4-week follow-up (*t*_2_; Figure 1). Randomization takes place after baseline assessment. Hence, the full duration of this study’s period for participants will be approximately 3.5 months (depending on the time between informed consent and completion of baseline assessment). Participants can withdraw consent from participation at any time. Participation will be terminated prematurely if participants are at risk for harm to themselves or others.

### Participants

#### Recruitment

Participants will include young people aged 14-25 years with climate change–related distress, who will be recruited from the general population via social media, youth counseling and social services, youth centers, food banks, schools, universities, youth organizations, and climate change– and sustainability-related organizations, groups, and institutions in Mannheim and the Rhine-Neckar region. While recruitment efforts are focused on this region, participants from outside this region can be included. All potential participants will be contacted by the research team, receive full information about this study, participate in a prescreening phone call, and, if they pass the initial screen, complete a full screening for eligibility. Participant appointments can take place on-site or via a certified and encrypted videoconferencing system.

#### Inclusion Criteria

Inclusion criteria are (1) aged 14-25 years, (2) having a score of ≥3.5 on the distress subscale and ≥2.3 on the impairment subscale on the Climate Change Distress and Impairment Scale (CC-DIS) [3]. CC-DIS cutoffs for inclusion represent the average scale mean of the 4 samples used in the validation study by Hepp et al [3]. The age range of 14-25 years is based on what has been widely used in the international youth mental health reform, recognizing that most mental health problems have their onset before the age of 25 years [40], as well as youth developmental considerations regarding the appropriateness of the training.

#### Exclusion Criteria

Young people are excluded from participation if they (1) report current treatment for or a current diagnosis of a severe mental disorder which they received from a professional (*ICD-10* [*International Statistical Classification of Diseases, Tenth Revision*] codes: F32.2, F32.3, F20, F22-29, F30.x, F60.3) or (2) report a significant reduction in functioning in the past 30 days (World Health Organization Disability Assessment Schedule 2.0 ≥41) [41]. The World Health Organization Disability Assessment Schedule 2.0 cutoff for exclusion is based on population norms, representing the 90th percentile on the 36-item version [41]. Other exclusion criteria comprise (3) indications of acute risk to self or others, (4) the inability to provide informed consent, and, in case of minors, no consent by parents or legal guardians, and (5) insufficient German language abilities, per assessment of the investigator.

### Intervention

#### Control Condition: CAU

Participants in the control condition will receive CAU, broadly defined, which includes access to all standard health care and social services. CAU broadly defined will include all public mental health services delivered according to local and national service guidelines and protocols by general practitioners, psychiatrists, clinical psychologists, school psychologists, social workers, psychological counselors, and other public mental health practitioners for mental health promotion, prevention, and treatment [42], available to participants in their living environment.

#### Experimental Condition: CliMACT+CAU

##### Overview

Participants allocated to the experimental condition receive the CliMACT training in addition to CAU. CliMACT will be delivered over a 6-week training period. It consists of an app-based, adaptive EMI and 3 sessions with a trained mental health professional, with a duration of 45-60 minutes, administered on-site or online using a certified and encrypted videoconferencing system. The EMI is geared toward real-time and real-world transfer of training content, principles, and techniques from face-to-face sessions to individuals’ daily lives using an app- and web-based system (InteractionDesigner platform, movisens GmbH). The CliMACT training is based on principles of CFI as well as ACT. Multimedia Appendix 2 provides an overview of the key training components during the 6-week training period. The EMI consists of 3 different delivery schemes of EMI components and EMA-based monitoring.

##### Enhancing EMI Delivery Scheme

During the training phase, participants are successively introduced to new EMI components based on CFI and ACT according to a set schema (see Multimedia Appendix 2; for example, after session 1, participants learn how to conduct specific breathing exercises). Participants can receive reminders for enhancing EMI components at self-set times.

##### Consolidating EMI Delivery Scheme

Participants are asked to practice EMI components that have already been introduced via the enhancing EMI delivery scheme on the following days. When all new tasks have been introduced (week 4), participants are encouraged to practice tasks randomly presented. Consolidating EMI components can be completed in response to reminders at self-set times or on demand.

##### Adaptive EMI Delivery Scheme

Participants’ responses to short, daily EMA questionnaires on momentary affect provide the basis for delivering CFI-based intervention via the adaptive EMI delivery scheme tailored to person, moment, and context. These are offered in moments of high negative affect, that is, when ratings exceed the threshold of an individual’s moving average on any negative affect item by ≥0.5 SDs. A prerequisite for the use of the moving average is that respondents have responded to at least 7 EMA prompts within the past 7 days. If this is not the case, a value of ≥4 on any negative affect item serves as a threshold for the adaptive EMI delivery scheme.

##### EMA-Based Monitoring and Feedback

CliMACT allows young people and mental health professionals to monitor young people’s well-being by delivering visual feedback in terms of reported climate change–related events, activities, affect, and completed training components, via the app and a web-based dashboard function.

The app further uses gamification elements to encourage practice and visualize progress throughout the 6 weeks. It provides FAQs (frequently asked questions) on training-relevant matters and web links to local and national climate change and sustainability-related topics and organizations to demonstrate network opportunities for collective action. The finalized CliMACT training manual, developed as part of the ADVANCE (Addressing Mental Health Vulnerabilities From Adolescence to Older Age: Innovating Prevention Science for Times of Change) project, will be published.

#### Adaptation

Theoretical and evidence-based considerations for interventions for young people with climate change–related distress informed adaptation from the original EMIcompass intervention to the CliMACT training (see Background section). Additionally, suggested adapted materials were reviewed with lived experience experts. Following recommendations from the World Health Organization’s Psychological Interventions Implementation Manual [43], cognitive interviews with 9 individuals with lived experience of subjective climate change–related distress were conducted to assess the understandability, acceptability, and relevance of the study materials (eg, visual design and written content). Feedback indicated good acceptability and primarily led to minor adjustments in wording, which were subsequently incorporated into the materials.

### Measures

#### Feasibility of Trial Methodology

The feasibility of trial methodology will be assessed based on the following criteria: feasibility of recruitment is assessed based on achieving the minimum monthly recruitment rate of 11 in (at least) the month with the highest recruitment rate of the feasibility RCT recruitment period. Feasibility of randomization is assessed based on achieving the minimum monthly rate of 10 participants successfully randomized after successful inclusion in at least 1 month. Retention is assessed using the percentage of retained participants at posttraining and 4-week follow-up.

#### Feasibility of Delivering the CliMACT Training

The feasibility of delivering the CliMACT training will be assessed at the posttraining assessment based on the criteria as delineated in the following. Satisfaction is measured through 3 scales. A 21-item participant debriefing questionnaire with a 7-point Likert scale ranging from 1-7, with higher values indicating higher satisfaction [16]. The 6-item perceived impact subscale of the Mobile App Rating Scale has a 6-point scale ranging from 0-5, with higher scores indicating higher perceived helpfulness. Lastly, there will be one item reflecting a global 5-star rating of the application, ranging from 1-5 [44].

Participant adherence is measured using the number of attended sessions and the number of completed consolidating and adaptive EMI components during training.

Fidelity to the training protocol is measured using a 19-item session component checklist adapted to CliMACT training components from the study by Reininghaus et al [16] with a yes-no format, and the 25-item Acceptance and Commitment Therapy Fidelity Measure with a 4-point scale ranging from 0-3 [45], with higher values indicating greater frequency of behavior aligned with ACT principles. Measures are completed by the mental health professional delivering the face-to-face session and by an independent rater. As active ingredients, CliMACT training components will be assessed as specific ingredients, and working alliance with the mental health professional and with the digital tool are measured as nonspecific ingredients, using the 12-item Working Alliance Inventory with a 5-point Likert scale ranging from 1-5 (Working Alliance Inventory-Self Report [WAI-SR]) [46,47] and 25-item Mobile Agnew Relationship Measure with a 7-point Likert scale ranging from 1-7 [48], with higher scores indicating a more positive working alliance for both measures.

#### Candidate Outcomes

As candidate outcomes, participants will complete the following self-report measures at baseline, post training, and 4-week follow-up: mental well-being is measured using the Warwick-Edinburgh Mental Well-Being Scale with 14 items using a 5-point Likert scale ranging from 1-5 [49,50]. The total score is a sum score, with higher scores indicating better mental well-being. Quality of life is measured using the World Health Organization Quality of Life measure with 26 items using a 5-point Likert scale ranging from 1-5 [51,52]. In the current study, the subscales for psychological health (6 items) and social relationships (3 items) will be considered as candidate outcomes. Domain scores are transformed to a scale from 0-100, with higher values indicating a higher quality of life. Subjective happiness is measured using the Subjective Happiness Scale with 4 items using a 7-point Likert scale ranging from 1-7 [53,54]. Reversing one negatively formulated item, a mean score is calculated, with higher scores indicating higher happiness. Climate change distress, used both as a screening and an outcome measure, is measured using the CC-DIS, with 15 items reflecting distress and 8 items reflecting impairment, using a 5-point Likert scale ranging from 1-5 [3]. After reversing items that indicate low levels of distress or impairment, a mean score is calculated, with higher scores indicating higher distress and impairment. General psychological distress is measured using the Core 10, a 10-item measure using a 5-point Likert scale ranging from 0-4 [55] and the Brief Symptom Inventory, a 53-item measure using a 5-point Likert scale ranging from 0-4 [56,57], with higher mean scores indicating higher psychological distress.

Using EMA (movisensXS app, movisens GmbH), 18 items are used to assess momentary affect on a Visual Analog Scale (VAS) ranging from 0-100. In this study, the mean of 4 unipolar positive affect facet items will be used as the total positive affect score. The mean of 9 unipolar negative affect items will be used as the total negative affect score. Based on the CC-DIS [3], 6 items are used to measure momentary climate change distress (3 items) and impairment (3 items), using a 7-point Likert scale ranging from 1-7. Mean scores will be used as scale scores. During the training phase, participants in the experimental condition complete an abbreviated EMA form in the InteractionDesigner platform, including 11 items assessing momentary affect on a 7-point Likert scale and a multi-select item to indicate which specific affects are currently related to climate change. All EMA items can be found in Multimedia Appendix 3.

#### Candidate Mechanisms

As candidate mechanisms, participants will complete the following measures at baseline, post training, and 4-week follow-up: self-efficacy is measured using the General Self-Efficacy Scale with 10 items using a 4-point scale ranging from 1-4 [58,59]. A sum score is calculated, with higher scores indicating higher levels of self-efficacy. Emotion regulation is measured using the Cognitive Emotion Regulation Questionnaire with 18 items using a 5-point Likert scale ranging from 1-5 [60,61]. First, subscale items are summed. Next, a mean score of 5 subscales is calculated for the adaptive emotion regulation scale, and a mean score of 4 subscales is created for the maladaptive emotion regulation scale. Resilience is measured using the Connor-Davidson Resilience Scale with 25 items using a 5-point Likert scale ranging from 0-4 [62,63]. The total score is a sum score, with higher scores indicating higher resilience. Self-compassion is measured using the Self-Compassion Scale with 26 items using a 5-point Likert scale, ranging from 1-5 [64,65]. Negatively formulated items are reversed, means for subscales calculated, and then subscale scores summed for the total score, with higher scores indicating higher levels of self-compassion. Mindfulness is measured using the Five-Facet Mindfulness Questionnaire with 24 items using a 5-point Likert scale ranging from 1-5 [66,67]. Facet scores are computed as sum scores (observing: 4 items; describing: 5 items, acting with awareness: 5 items; nonjudging of inner experience: 5 items, and nonreactivity to inner experience: 5 items. Psychological inflexibility is measured using the Acceptance and Action Questionnaire II with 7 items using a 7-point Likert scale ranging from 1-7 [68,69]. The total score is a sum score, with higher scores indicating higher psychological inflexibility. Internalized stigma is measured using an adapted version of the Internalized Stigma of Mental Illness scale with 29 items using a 4-point scale ranging from 1-4 [70,71]. The measure covers alienation (6 items), stereotype endorsement (7 items), discrimination experience (5 items), social withdrawal (6 items), and stigma resistance (5 items). A mean score is calculated as the total score, excluding the stigma resistance subscale [72]. Experiences of discrimination are measured with the adapted version of the major experiences of discrimination measure [73], using a multi-select item asking about the experience of unfair treatments in different areas (police, judicial system, discouraging education, neighbors and family, service, medical attention, public transport, school, superiors, and other) due to the participants’ climate change–related attitudes, views and actions. For areas in which unfair treatment is reported, participants can indicate the number of times this has occurred. The total number of experiences is used as the total score. Self-stigma of seeking help is measured using the Self-Stigma of Seeking Help Scale with 10 items using a 5-point Likert scale ranging from 1-5 [74]. Reversing positively phrased items, a sum score is used as the total score, with higher values indicating higher self-stigma. PEB is measured using an adapted and translated version of the PEB measure with 18 items using a VAS ranging from 0-100 [75]. Mean scores are calculated for personal behavior (10 items) and collective behavior (8 items), with higher scores indicating more opportunities at which the participant has engaged in PEBs.

Using EMA, momentary self-efficacy is measured with 2 items based on the measure per Majeed et al [76] on a VAS ranging from 0-100. Positive and negative event intensity are respectively measured on a 7-point Likert scale ranging from 1-7. For negative event intensity ≥2, momentary emotional resilience is assessed using 1 item [12] on a VAS ranging from 0-100. Current activity is reported using a multi-select item, and for each confirmed activity, 1 of 10 matching momentary PEBs is queried on a VAS ranging from 0-100. Momentary value-based living is measured using 3 adapted items from the Engaged Living Scale [77] on a VAS ranging from 0-100. During the training phase, activity and events, but not momentary self-efficacy, PEB, or value-based living, will be assessed in daily EMA.

#### Measures for the Economic Evaluation

Two measures for the economic evaluation will be administered to assess health-related quality of life, use of care services, and the associated costs of care and service use. Health-related quality of life is measured using the EQ-5D-Y-3L instrument with 5 items using a 3-point Likert scale, ranging from 1-3, with higher scores indicating lower health-related quality of life, and 1 VAS item ranging from 0-100, with higher scores indicating better health [78,79]. The information on use of care services, which is needed to calculate the associated costs of care service use, is captured using an adapted version of the Client Service Receipt Inventory (CSRI) [80,81]. Together with other self-report measures of the trial, both measures for the economic evaluation (EQ-5D-3L and CSRI) will be administered at the 3 time points: at baseline, post training, and 4-week follow-up.

#### Other Measures

**Figure 2:**
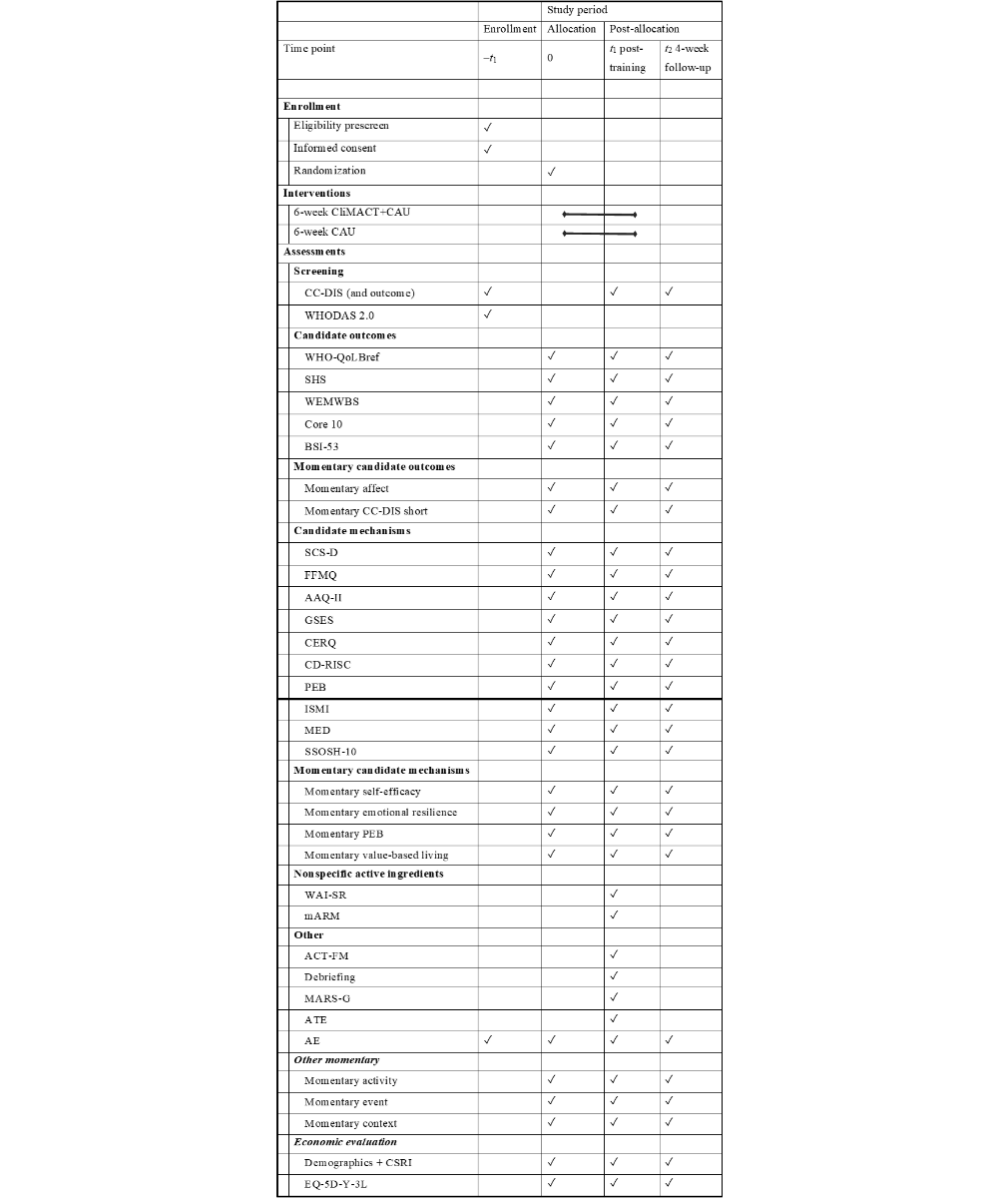
SPIRIT figure. AAQ-II: Acceptance and Action Questionnaire; ACT-FM: Acceptance and Commitment Therapy Fidelity Measure; AE: adverse events; ATE: adverse trial effects; BSI-53: Brief Symptom Inventory 53; CAU: care as usual; CC-DIS: Climate Change Distress and Impairment Scale; CD-RISC: Connor-Davidson Resilience Scale; CERQ: Cognitive Emotion Regulation Questionnaire; CliMACT+CAU: Climate - Mind and Act+Care as Usual; CSRI: Client Service Receipt Inventory; FFMQ: Five Facet Mindfulness Questionnaire; GSES: General Self Efficacy Scale; ISMI: Internalized Stigma of Mental Illness-Scale; mARM: Mobile Agnew Relationship Measure; MARS-G: Mobile App Rating Scale - German Version; MED: major experiences of discrimination; PEB: proenvironmental behavior; SCS-D: Self-Compassion Scale - German Version; SHS: Subjective Happiness Scale; SPIRIT: Standard Protocol Items: Recommendations for International Trials; SSOSH-10: Self-Stigma of Seeking Help; WAI-SR: Working Alliance Inventory - Self Report; WEMWBS: Warwick-Edinburgh Mental Well-Being Scale; WHO-QoLBref: World Health Organization Quality of Life - Brief Version; WHODAS 2.0: World Health Organization Disability Assessment Schedule 2.0.

At each assessment, it will be documented if any adverse events (AEs) related or unrelated to the training or study participation have occurred, including health-related events, technical problems with the smartphone, problems with the digital application, or other issues. Marginalized or vulnerable group status is reflected by equity-relevant sociodemographic indicators at baseline and coded present when at least one of the following is true: female gender, ethnic minority, migration history, low family SES, current urbanicity, and lower education level. At posttraining, adverse trial effects are measured with the measure by Hutton et al [82], using a 5-point Likert scale ranging from 1-5. An overview of all measures and time points can be found in Figure 2 (in line with the SPIRIT [Standard Protocol Items: Recommendations for Interventional Trials] statement [83]).

### Randomization and Blinding

After completing baseline assessments, participants will be randomized at a 1:1 ratio to the experimental or control condition. Randomization will be performed by a nonblinded researcher training delivery team through a computer-generated sequence not accessible to blinded team members, applying block randomization with blocks of 5. Nonblinded researchers inform participants of their allocation. All outcomes will be assessed, and the statistical analysis will be performed, blind to random allocation. If unblinding occurs, this will be documented, and a different study team member will continue follow-up assessment for the respective participant.

In the experimental condition, a microrandomized trial (MRT) with repeated within-person randomization is implemented in the adaptive EMI components. Six CFI-based EMI components become available throughout weeks 1-3. If participants exceed the negative affect threshold (within-subject mean ±0.5 SDs) for triggering adaptive EMI components, participants will be randomized to receive a CFI-based EMI component they are already familiar with (ie, components that have been introduced as an enhancing EMI component) as experimental condition or no EMI component as control condition with a randomization ratio of 6:1. Repeated within-person randomization will be carried out via the InteractionDesigner platform.

### Justification of Sample Size

#### Feasibility RCT

The sample size for this feasibility RCT is primarily determined to address the primary objective to provide the basis for successful recruitment in a fully powered efficacy-effectiveness RCT. Initial sample size planning points to ≈180 participants required for the full RCT. To this end, we set a feasibility limit of a minimum monthly recruitment rate of 11 participants in (at least) the month with the highest recruitment rate of the feasibility trial recruitment period. This lower bound reflects the minimum monthly recruitment rate required to recruit an expected ≈180 participants for a subsequent fully powered RCT within a designated period of 17 months. This study’s population for the feasibility RCT will comprise a maximum of 50 youth aged 14-25 years (n=25 in the experimental and control conditions, respectively) with climate change–related distress. This upper limit of 50 is based on recommendations by Whitehead et al [84] for determining sample size for feasibility RCTs and would reflect a monthly recruitment rate of 17 if recruitment can be completed within 3 months. Thus, the feasibility trial will inform us about the feasibility of the aspired recruitment rate and the possibility of overrecruitment.

#### MRT Sample Size Calculation

To address the objective of exploring CliMACT training components as specific active ingredients, an MRT will be conducted. Based on the power approximation for MRTs [85,86], a sample size of 25 participants is sufficient to detect a small effect size of *d* ≥0.15 on a proximal EMA outcome, with *t*=252 decision points (based on our EMIs with a training duration of 42 days with 6 random EMA prompts per day) and a power of 80% for a 2-sided test α=.05, with an availability rate η (ie, proportion of completed EMA observations at given decision point T) of 50% and a randomization probability of 0.85 (ie, a ratio of 6:1 for experimental vs control components).

### Safety Considerations

All AEs or serious adverse events (SAEs) will be monitored and collected throughout this study’s period. SAEs include any serious incidents resulting in death, persistent or significant disability or incapacity, requiring (extension of) hospitalization, or life-threatening situations. AEs include any nonserious incidents regarding the health and well-being of the participant, related or unrelated to trial participation, as well as technical problems with the hardware (ie, study smartphone), software (ie, smartphone app and assessments), and training used in this trial. SAEs are not expected to occur as a result of the intervention or study participation. If occurring, SAEs will be reported to this trial’s steering committee (TSC) and ethics and data advisory board (EDAB), which can advise on ethical concerns and monitor evidence for intervention harm. At the end of this trial, SAE will be reported to the local ethics committee. AE and SAE will be reported in publications of this study’s results. Insurance is in place for participants who endure harm through their participation in this study.

### Statistical Analysis

#### Feasibility

All statistical analyses will be performed using Stata 17 (StataCorp LLC). To address the objectives of establishing the feasibility of this trial’s methodology and delivery of the CliMACT training, descriptive statistics will be used (mean, SD, n, %). Evaluation of meeting important feasibility criteria will be based on a traffic light system with 3 categories, with green indicating that full feasibility is established, yellow indicating that feasibility is established, but study procedures need to be modified, and red indicating that feasibility is not established. Feasibility of trial methodology (objective 1) will be based on recruitment, randomization, and retention, and evaluated according to a priori determined cutoffs shown in Table 1, with the primary objective of establishing the feasibility of recruitment for a future efficacy-effectiveness RCT (that informed our justification of the sample size, see above). Feasibility of delivering the CliMACT training (objective 2) will be based on participant satisfaction, participant adherence, and mental health professional fidelity to the training protocol, and will also be evaluated according to predetermined cutoffs (Table 1).

**Table 1:** Traffic light criteria for the feasibility of trial methodology and the feasibility of delivering the CliMACT^a^ training.

			Green		Yellow		Red
**Feasibility of trial methodology**							
	Recruitment	Monthly recruitment rate of n=11 to n=17 participants in (at least) the month with the highest recruitment rate during the feasibility RCT^b^ recruitment period		Monthly recruitment rate of n=5 to n=10 participants in (at least) the month with the highest recruitment rate during the feasibility RCT recruitment period; recruitment area should be opened to places outside Mannheim and Rhine-Neckar		A monthly recruitment of n<5 participants for all months of the feasibility RCT recruitment period	
	Randomization	Successful randomization of n=10 to n=17 participants in (at least) the month with the highest randomization rate during the feasibility RCT recruitment period		Successful randomization of n=4 to n=9 participants in (at least) the month with the highest randomization rate of the feasibility RCT recruitment period		Successful randomization of <4 participants in all months of the feasibility RCT recruitment period	
	Retention	Retention rate of at least 85% of all randomized participants, for outcome assessment of at least one of the assessment time points at posttraining and 4-week follow-up		Retention rate of at least 75% for outcome assessment of at least one of the assessment time points at posttraining and 4-week follow-up		<75% retention at both posttraining and 4-week follow-up	
**Feasibility of delivering the CliMACT training**							
	Participants satisfaction with the training	Moderate to high satisfaction in general in a debriefing questionnaire (mean score of ≥4 on a 7-point scale) and the global star-rating of the Mobile Application Rating Scale (mean rating of ≥3 on a 5-point scale)		Fair satisfaction in general in a debriefing questionnaire (mean rating of >3 and <4) and the global star-rating of the Mobile Application Rating Scale (mean rating of >2 and ≤3). Adjustment and optimization of the training or app are required		Low satisfaction in general in the debriefing questionnaire (mean rating ≤3) and the global star-rating of the Mobile Application Rating Scale (mean rating ≤2)	
	Participant adherence to intervention protocol	Moderate to strong adherence to training sessions (at least 80% of participants attending at least 2 of the 3 face-to-face sessions) and EMI^c^ components (mean of ≥1 consolidating or adaptive EMI components completed per week)		Fair adherence to training sessions (at least 60% of participants attending at least 2 of the 3 face-to-face sessions) and EMI components (mean of 0.8 consolidating or adaptive EMI components completed per week). Adjustment and optimization of sessions or the app are required		Poor adherence to training sessions (<60% of participants completing at least 2 of the 3 face-to-face sessions) and EMI components (mean of <0.8 consolidating or adaptive EMI components per week)	
	Mental health professional fidelity to training protocol	Moderate to high fidelity to intervention protocol (≥80% of core components delivered by trained psychologist) and use of ACT^d^-consistent skills (mean rating of ≥1.5 on a 4-point scale ranging from 0-3)		Fair fidelity to intervention protocol (≥60% of core components delivered by trained psychologist) and use of ACT-consistent skills (mean rating of >1 and <1.5 on a 4-point scale ranging from 0-3). Adjustment of sessions, EMI app, or training of psychologists is required		Poor fidelity to intervention protocol (<60% of core components delivered) and use of ACT-consistent skills (mean rating of <1 on a 4-point scale ranging from 0-3)	

^a^CliMACT: Climate Mind and Act.

^b^RCT: randomized controlled trial.

^c^EMI: ecological momentary intervention.

^d^ACT: acceptance and commitment therapy.

As nonspecific active ingredients (objective 2 continued), we will explore the association of working alliance toward mental health professionals as well as working alliance with the app-based EMI with the 8 candidate outcomes in participants of the experimental condition, using linear mixed models. Separate models will be fitted for each alliance type and candidate outcome at both posttraining and 4-week follow-up; additionally, time (posttraining and 4-week follow-up), the respective outcome at baseline, and working alliance will be entered as independent variables (details set in preregistered statistical analysis plan, becoming public on OSF after publication of results) [87]. A level-2 random intercept for subject and a random effect for time will be added, and level-1 residuals will be allowed to be correlated to account for within-subject nesting of repeated measures. For the 2 momentary outcomes of nonspecific active ingredients, outcomes measured at baseline will be person-mean centered, and, in addition to the above specifications, random slopes for time and working alliance will be added, with an unstructured variance-covariance matrix. Within-subject residuals will be modeled with an autoregressive structure of the exponential type, allowing the models to account for unequally spaced time values. Restricted maximum likelihood estimation will be applied.

As specific active ingredients (objective 2 continued), we will explore proximal effects of CFI-based CliMACT training components on momentary negative affect in an MRT design in participants in the experimental condition using linear mixed modeling. MRT condition (experimental: any adaptive CFI-based training component vs control: no adaptive training component, randomized at a ratio of 6:1 in moments of higher negative affect), negative affect at *t*_n_ (person-mean centered), and time between assessment time points will be entered as independent variables. Negative affect at *t*_n+1_ will be entered as the dependent variable. A level-2 random intercept will be added, and level-1 residuals will be allowed to be correlated to account for within-subject nesting of repeated measures. Restricted maximum likelihood estimation will be applied.

#### Initial Signals of Efficacy on Candidate Outcomes and Mechanisms

Initial signals of efficacy of the CliMACT training will be explored according to the intention-to-treat principle (ie, data of all participants randomized to 1 of the 2 conditions will be entered into the analysis). Before data analysis, all data will be checked for quality and plausibility. For all analyses, we will inspect the 95% CI and obtain d-type effect sizes given their exploratory nature. Linear mixed modeling will be used to compare candidate outcomes (objective 3) and candidate mechanisms (objective 4) between the experimental and control conditions at posttraining and 4-week follow-up. Each candidate outcome and mechanism (applying joint analyses to posttraining and 4-week follow-up assessment) will be used as a dependent variable in separate analyses. Time (posttraining and 4-week follow-up), condition (experimental and control condition), and the respective outcome measured at baseline (grand-mean centered) will be entered as independent variables. A level-2 random intercept will be added, and level-1 residuals will be allowed to be correlated to account for within-subject nesting of repeated measures. Restricted maximum likelihood estimation will be applied. The coefficient for the condition reflects the difference between the intervention and control arm for the post- and 4-week follow-up assessments jointly. We will next extend the model by a condition × time interaction to determine the time-specific contrasts, reflecting differences between the 2 conditions at each time point, and an outcome or mechanism at baseline × time interaction.

Model specifications for momentary outcomes and mechanisms are as follows. The respective putative mechanism (momentary value-based living, momentary emotional resilience, momentary self-efficacy, and momentary PEB) or outcome (positive affect, negative affect, climate change distress, and climate change impairment) as measured 8 times per day on 6 consecutive days will be entered as dependent variables in separate models. As independent variables, the respective candidate EMA-based mechanism or outcome at baseline (person-mean-centered), time, condition, a mechanism or outcome at baseline × time interaction, and a condition × time interaction are entered. A 2-level model will be estimated with time points nested in participants. Random intercepts will be included for participant and time point. Random slopes will be included for time and the respective candidate mechanism or outcome, applying an unstructured variance-covariance matrix. For EMA observations at each time point (posttraining and 4-week follow-up), we assume within-subject residuals to be autocorrelated and apply an autoregressive structure of the exponential type. Hours since midnight of the first EMA day are used as a time indicator in our models. This approach, in contrast to using the beep number, accounts for unequally spaced delays between beeps. Again, restricted maximum likelihood estimation will be applied. Next, the respective model will, again, be extended by a condition × time interaction in order to determine the time-specific contrasts, reflecting differences between the 2 conditions at each time point.

#### Subgroup Analyses by Marginalized/Vulnerable Group Status

To address the objective of examining the feasibility of establishing credibility criteria for a priori planned subgroup analyses (objective 5), we will explore the feasibility of generating a marginalized/vulnerable group status score by, first, descriptively exploring the distribution of indicators for health inequities relevant to climate change–related distress as a basis for subgroup analyses (family SES, level of education, sex, migrant status, ethnic minority status, and current urbanicity) in the sample. Second, descriptively exploring the distribution of a combined marginalized/vulnerable group status variable (indicated by any of the following characteristics: family SES=low, level of education=lower, gender≠male, migrant status=yes, ethnic minority status=yes, and current urbanicity=yes) and a marginalized group status variable (indicated by any of the following characteristics: family SES=low, level of education=lower, migrant status=yes, ethnic minority status=yes; thus excluding gender and current urbanicity in comparison to the combined marginalized/vulnerable group status variable). Third, conducting subgroup analyses on candidate outcomes stratified by combined marginalized/vulnerable group status (yes or no) and by marginalized group status (yes or no), as defined in the second point above.

We will use a restricted set of established credibility criteria for subgroup analyses as appropriate for this feasibility RCT [88]: (1) measuring indicators for health inequities relevant to climate change–related distress at baseline, that is, before randomization, and (2) exploring differences in effect sizes and CIs stratified by subgroups with and without marginalized/vulnerable group status.

#### Feasibility of Economic Evaluation

For the report of service use in the CSRI and the report of health-related quality of life in the EQ-5D-Y-3L, we will report missing rates and descriptive statistics (mean, SD, range, n, %). Response patterns will inform the handling of variables, possible adaptation of items or response categories, and statistical methodology of the economic evaluation in an efficacy-effectiveness RCT. Furthermore, we will explore the distribution of cost patterns across experimental and control groups to gain a first insight into differences between the 2 groups.

### Research Governance

This study is part of the ADVANCE consortium, coordinated by the University of Copenhagen. The Central Institute of Mental Health is the lead of this feasibility RCT. Amendments to this study’s protocol will be submitted to the local ethics committee for approval and communicated to relevant parties (eg, TSC, EDAB, funder, and collaborating institutions) and will be updated in this trial’s registry. The principal investigator has overall responsibility for this trial. This trial coordinator and research team are responsible for the day-to-day management of this trial, monitoring trial progress (recruitment, assessment, and training delivery), and preparation of reports to the ethics committee, TSC, and EDAB. The TSC will consist of the trial’s statistician and the SAG, and will be consulted biannually in a joint meeting with the research team, providing advice on cocreative practical, scientific, and target-group related matters. The project consortium holds monthly research meetings, as well as a quarterly management meeting, including EDAB as a standing agenda item. The EDAB will offer guidance, advice, and recommendations on four main aspects: (1) data management, protection, and privacy; (2) recruitment, inclusion, and exclusion criteria, and informed consent procedures; (3) improvement of the project data management and ethics plans; and (4) adherence to European Union regulations on ethical research trials. The EDAB will advise on any specific ethical question arising during the project, including any issues concerning the safety, rights, and well-being of study participants and research teams (including serious AEs in the clinical trials), as well as concerns regarding incidental study findings or tensions in reporting and dissemination of potentially contentious research findings. The Scientific Advisory Board (SAB) is an external advisory body of the ADVANCE consortium that aims to enhance the quality and impact of the project. The SAB will (1) serve as a resource for knowledge and expertise; (2) advise the consortium on political, social, environmental, technological, legal, and economic factors that may influence the project; and (3) give advice on the project’s interaction with related research projects and initiatives. The SAB participates in project meetings, videoconferences, and workshops of the ADVANCE consortium as appropriate.

### Ethical Considerations

This study has received ethical approval from the local ethics committee (Medical Faculty Mannheim, Heidelberg University, 2024-597). All participants (and in case of minors, their legal guardians) will sign written informed consent and receive financial compensation of up to € 110 Euro(US $145.60) for participation. Minors give written assent. Research data will be handled in compliance with the German and European Data Protection Regulation. Data will be stored securely and pseudonymized; that is, research data will be stored using a number code. Personal data will be kept separately from pseudonymized data. Participants who withdraw from this study can decide whether data collected up to that date can be processed or should be destroyed.

## Results

Cocreation of this study design started in November 2023. Adaptation of the EMI to meet the needs of young people with climate change–related distress took place between February and September 2024. The trial was registered at ISRCTN (no. ISRCTN33613914) in November 2024. All study procedures were approved by the local ethics committee in September 2024 (Amendment I: November 2024). Recruitment started in November 2024, and the first enrollment was in December 2024. Data collection was completed by August 25, 2025. The results from the feasibility RCT are expected to be published in 2026. Its findings are going to inform the methodology of a fully powered efficacy-effectiveness RCT.

## Discussion

As the main objective, this study will provide evidence on the feasibility of trial methodology (based on recruitment, randomization, and retention) as well as the feasibility of delivering the CliMACT training (based on satisfaction, compliance, and fidelity with the training manual). In addition, it will provide the first evidence on initial signals of efficacy of the CliMACT training on candidate outcomes and mechanisms. Climate change–related distress is common in youth [2,4]. To date, strategies for mental health promotion and prevention in this population are limited. Especially, evidence-based strategies to support this population using EMIs tailored to person, moment, and context are currently lacking. Therefore, the primary aim of this study will be to evaluate the feasibility of a hybrid adaptive EMI for mental health promotion in young people with climate change–related distress. The approach that we adopt here focuses on the ecological translation of CFI-based and ACT-based principles to the daily lives of young people with climate change–related distress.

Based on findings on feasibility of trial methodology from the current feasibility RCT, we will decide on necessary adaptations about recruitment sites and strategies (eg, moving from local to nationwide recruitment), the randomization procedure (eg, exposing errors), and strategies to maintain or improve retention (eg, optimize documentation, notification of participants, incentives, and reduction of number of measures). Based on findings on the feasibility of delivering the CliMACT training, we will improve intervention delivery based on quantitative and qualitative data on face-to-face sessions and EMI (eg, training components, frequency of EMAs, and visualizations), and optimize acceptability of the training in a future efficacy-effectiveness RCT. Initial signals of efficacy from the feasibility RCT will, based on effect size estimates, support the selection of a primary outcome for an efficacy-effectiveness RCT and will help us to formulate a more accurate theory of change model, which will be tested in the future.

Digital interventions for youth provide the benefit of being highly accessible and scalable. Recent evidence suggests that youth have generally positive attitudes toward mental health apps [89,90]. Meta-analytic evidence suggests that human support and guidance are central for digital mental health interventions to be effective [91]. Recent findings on the feasibility and efficacy of hybrid EMIs in youth [15,16] further support a guided approach. Based on this evidence, CliMACT was designed and developed adopting a hybrid, adaptive EMI. This was also informed by cocreation activities with lived experience experts and stakeholders at the outset of this study, opting for a hybrid rather than a stand-alone digital approach to ensure participants’ understanding of training principles and foster adherence. In addition, prior research suggests young people prefer digital mental health interventions that contain videos, limited text, options for personalization, the ability to connect with others, and text message reminders [92]. Building on these findings, using a chat-based format, providing audio and video guides for EMI components, and the opportunity to implement personalized time windows for EMA prompts and reminders for EMI components, our cocreation and adaptation work has addressed several of these aspects in developing the CliMACT training. At the outset, SAG and scenario-based stakeholder workshops advised to primarily address a broader target group of young people with climate change–related distress, who would benefit from a generic training for mental health promotion, rather than prevention and early intervention for a smaller subgroup with clinically relevant symptoms. Hence, tailoring CliMACT to more specific target groups (eg, individuals affected by extreme weather events, climate activists, and individuals with pre-existing conditions) remains to be addressed by future research.

If the feasibility of the trial methodology and intervention delivery can be successfully established in the present feasibility RCT, this will provide the basis for a fully powered efficacy-effectiveness RCT. Findings from the feasibility RCT will be discussed with the SAG as a basis for cocreating adaptations before the efficacy-effectiveness RCT. As such, the feasibility RCT will directly inform the methodology of, and intervention delivery in, the fully-powered RCT.

EMI designs provide methodological advantages related to improving our understanding of the underlying mechanisms of mental health difficulties as well as the efficacy and effectiveness of interventions, building on an ecological interventionist causal model approach [11]. This approach allows us to establish criteria relevant to sole plausibility, such as association and temporal order. Sole plausibility can be addressed by examining whether an intervention component can modify a proposed mechanism in daily life, and whether changes in the proposed mechanism are, in turn, associated with changes in the outcome. This is what we set out to address in our future efficacy-effectiveness RCT.

In line with the broad scope of mental health promotion in public mental health provision, the comparator condition was selected to be CAU, broadly defined, as the systematic delivery of evidence-based strategies for mental health promotion in the broader public mental health care domain is still limited. Therefore, a key question from a public health (and service commissioner) perspective remains whether CliMACT confers superiority beyond that of broad CAU. Hence, design and comparator in this trial (ie, CAU, broadly defined) reflect an optimal choice for ensuring high external and ecological validity, as it mirrors the naturalistic service landscape and practice to which beneficial effects ought to generalize, including to newly emerging digital public mental health services.

This study will provide the first evidence of the feasibility and initial signals of efficacy of CliMACT, a hybrid, adaptive EMI targeted specifically at young people with climate change–related distress. While this study’s design has considerable strengths (including cocreation, predefined feasibility criteria, randomization and blinding, an MRT, and assessment of equity-related participants’ characteristics), several limitations need to be considered before interpreting findings from this feasibility RCT. While findings will support the selection of the primary outcome of CliMACT and develop the theory of change of the training further, uncertainty will remain in effect size estimates that will be minimized for the primary outcome by the fully powered RCT. Nevertheless, given the lack of prior empirical data on interventions in the target group, this feasibility RCT will lay important groundwork. Next, we are going to carefully evaluate the potential burden for participants due to the number and length of measures. Based on the feasibility RCT, we will identify and prioritize measures for which sensitivity to change is supported by efficacy signals and improve the selection of measures accordingly. Last, while individual-based interventions bear substantial potential and have their rightful place, it is important to note that climate change is a societal and global challenge rather than an individual challenge. Hence, developing strategies to support individuals’ emotional resilience to climate change must be paralleled by, and not replace, collective and political action. The need for community-level strategies is currently endorsed by conceptual work on interventions addressing climate change–related distress at this level [17-19]. The CliMACT training, for now, addresses community building by means of suggestions for network opportunities. On a different note, it remains a challenge for digital mental health research to establish whether and how digital interventions actually offer effective support and increase accessibility for marginalized groups, with currently moderate evidence and further need for high-quality studies [93]. In the current study, we will establish whether the current trial and training methodology allows for reaching a sufficient proportion of young people in marginalized or vulnerable situations as a basis for evaluating the equity of CliMACT in our future work. Monitoring key sociodemographic indicators in intervention research with regard to reach and effectiveness is crucial to work toward interventions that mitigate, rather than augment, mental health disparities [33]. Overall, feasibility as established in the current study, coupled with efficacy and effectiveness that are to be fully established in a future efficacy-effectiveness RCT, lay the foundation for potential real-world implementation of accessible and equitable measures for mental health promotion, such as CliMACT as a relevant public mental health strategy. Trial results will be communicated to participants, lay audiences (traditional and social media coverage), stakeholders (SAG members and their networks), and researchers (national and international conferences and peer-reviewed journals).

## Data Availability

The datasets generated and analyzed during this study are available from the corresponding author upon reasonable request.

## Appendix

Multimedia Appendix 1 SPIRIT 2025 checklist of items to address in a randomized trial protocol.

Multimedia Appendix 2 Key components of the CliMACT ecological momentary intervention.

Multimedia Appendix 3 EMA items (English). EMA: ecological momentary assessment.

## Abbreviations

ACT: acceptance and commitment therapy
ADVANCE: Addressing Mental Health Vulnerabilities From Adolescence to Older Age: Innovating Prevention Science for Times of Change
AE: adverse event
CAU: care as usual
CC-DIS: Climate Change Distress and Impairment Scale
CFI: compassion-focused intervention
CliMACT: Climate Mind and Act
CONSORT: Consolidated Standards of Reporting Trials
CSRI: Client Service Receipt Inventory
EDAB: ethics and data advisory board
EMA: ecological momentary assessment
EMI: ecological momentary intervention
FAQ: Frequently Asked Question
ICD-10: International Statistical Classification of Diseases, Tenth Revision
MRT: microrandomized trial
PEB: proenvironmental behavior
PROGRESS: Place of Residence; Race, Ethnicity, Culture, or Language; Occupation; Gender or Sex; Religion; Education; Socioeconomic Status; and Social Capital
RCT: randomized controlled trial
SAB: Scientific Advisory Board
SAE: serious adverse event
SAG: society advisory group
SES: socioeconomic status
SPIRIT: Standard Protocol Items: Recommendations for Interventional Trials
TSC: trial steering committee
VAS: Visual Analog Scale
WAI-SR: Working Alliance Inventory-Self Report
