# Supplementary material for: Digital Training for Mental Health Promotion in Young People With Climate Change-Related Distress: Protocol for a Feasibility Randomized Controlled Trial
Source: JMIR Res Protoc. 2025 Dec 5;14:e77764. doi: 10.2196/77764 (PMC12717510; doi:10.2196/77764)
Supplement: Multimedia Appendix 2 [file resprot_v14i1e77764_app2.docx]

**Table S1**. Key components of the CliMACT ecological momentary intervention.

| CliMACT training (weeks) |  |  |  |  |  |  |
| --- | --- | --- | --- | --- | --- | --- |
|  | 1 | 2 | 3 | 4 | 5 | 6 |
| Face-to-Face sessions | Session 1: introduction CliMACT, climate emotions, CFI-based EMI components | Session 2: review of experiences and monitoring, introduction values and value-based EMI component |  | Session 3: review of experiences and monitoring, introduction obstacles and helpful strategies |  |  |
| Enhancing EMI components | Counting breath  +  Breathing with pause | Safe place  +  Emotion as a wave  +  Values & value-based actions | Compassionate companion  +  Compassionate self | Identifying obstacles and helpful strategies |  |  |
| Consolidating EMI components | Practice once per day for 1-5min one of the following components:   - Counting breath - Breathing with pause | Practice once per day for 1-5min one of the following components:   - Safe place or - Emotion as a wave - Morning: Daily - planned value-based action - Evening evaluation: How satisfied with planned action VAS 0-100? | Practice once per day for 1-5min one of the following components:   - Compassionate companion - Compassionate self - Morning: Weekly - extension of personal values; Daily - planned value-based action - Evening evaluation: How satisfied with planned action VAS 0-100? | Practice once per day for 1-5min one of the following components, randomly assigned:   - Counting breath - Breathing with pause - Safe place - Emotion as a wave - Compassionate companion - Compassionate self - Morning: Weekly - extension of personal values; Daily - planned value-based action - Evening evaluation: How satisfied with planned action VAS 0-100? | Practice once per day for 1-5min one of the following components randomly assigned:   - Counting breath - Breathing with pause - Safe place - Emotion as a wave - Compassionate companion - Compassionate self - Morning: Weekly - extension of personal values; Daily - planned value-based action - Evening evaluation: How satisfied with planned action VAS 0-100? | Practice once per day for 1-5min one of the following components, randomly assigned:   - Counting breath - Breathing with pause - Safe place - Emotion as a wave - Compassionate companion - Compassionate self - Morning: Weekly - extension of personal values; Daily - planned value-based action - Evening evaluation: How satisfied with planned action VAS 0-100? |
| Adaptive EMI components | Randomly assigned in moments of higher negative affect:   - Counting breath - Breathing with pause | Randomly assigned in moments of higher negative affect:   - Counting breath - Breathing with pause - Safe place - Emotion as a wave | Randomly assigned in moments of higher negative affect:   - Counting breath - Breathing with pause - Safe place - Emotion as a wave - Compassionate companion - Compassionate self | Randomly assigned in moments of higher negative affect :   - Counting breath - Breathing with pause - Safe place - Emotion as a wave - Compassionate companion - Compassionate self   Evening: if consolidating VAS<50: obstacle & helpful strategies | Randomly assigned in moments of higher negative affect:   - Counting breath - Breathing with pause - Safe place - Emotion as a wave - Compassionate companion - Compassionate self   Evening: if consolidating VAS<50: obstacle & helpful strategies | Randomly assigned in moments of higher negative affect:   - Counting breath - Breathing with pause - Safe place - Emotion as a wave - Compassionate companion - Compassionate self   Evening: if consolidating VAS<50: obstacle & helpful strategies |

Note. CFI = Compassion focused intervention. VAS = Visual analogue scale. For enhancing CFI-based EMI components, participants receive a long instruction with extensive guidance. For consolidating and adaptive CFI-based EMI components, participants can choose between the long instruction for more guidance, and a shorter instruction with less guidance or a helpful animation. CFI-based EMI components and daily planned value-based action, once available as consolidating component, can always be completed on demand.
