# Supplementary material for: Digital Training for Mental Health Promotion in Young People With Climate Change-Related Distress: Protocol for a Feasibility Randomized Controlled Trial
Source: JMIR Res Protoc. 2025 Dec 5;14:e77764. doi: 10.2196/77764 (PMC12717510; doi:10.2196/77764)
Supplement: Multimedia Appendix 3 [file resprot_v14i1e77764_app3.docx]

| **Concept** | Item | Format | Scale |
| --- | --- | --- | --- |
| **Affect** | At the moment I feel... | introduction text |  |
|  | good | VAS | Not at all; very (0-100) |
|  | bad | VAS | Not at all; very (0-100) |
|  | unwell-well | VAS | bipolar (0-100) |
|  | dissatisfied-satisfied | VAS | bipolar (0-100) |
|  | tired-awake | VAS | bipolar (0-100) |
|  | At the moment I feel... | introduction text |  |
|  | active | VAS | not at all; fully and completely (0-100) |
|  | happy | VAS | not at all; fully and completely (0-100) |
|  | confident | VAS | not at all; fully and completely (0-100) |
|  | relaxed | VAS | not at all; fully and completely (0-100) |
|  | downhearted | VAS | not at all; fully and completely (0-100) |
|  | upset | VAS | not at all; fully and completely (0-100) |
|  | afraid | VAS | not at all; fully and completely (0-100) |
|  | sad | VAS | not at all; fully and completely (0-100) |
|  | guilty | VAS | not at all; fully and completely (0-100) |
|  | helpless | VAS | not at all; fully and completely (0-100) |
|  | lonely | VAS | not at all; fully and completely (0-100) |
|  | nervous | VAS | not at all; fully and completely (0-100) |
|  | stressed | VAS | not at all; fully and completely (0-100) |
| **CCDI short** | Since the last query I have been ... |  |  |
|  | angry about how little is being done to combat climate change. | Likert | 1 = not at all, 7 = very strongly |
|  | worried about the future because I'm thinking about climate change. | Likert | 1 = not at all, 7 = very strongly |
|  | sad because of the impact that climate change is having on the planet | Likert | 1 = not at all, 7 = very strongly |
|  | overwhelmed by everyday life due to climate change. | Likert | 1 = not at all, 7 = very strongly |
|  | impaired in my relationships because of discussions about climate change . | Likert | 1 = not at all, 7 = very strongly |
|  | unable to get myself to work/study because I was thinking about climate change | Likert | 1 = not at all, 7 = very strongly |
| **Social context** | Who am I with? | select one | 1 = alone  2 = together with others  3 = online/on the phone with others |
|  | I am with… | Select many | 1 = my partner  2 = my family, my relatives  3 = a friend, some friends  4 = colleagues, classmates, fellow students  5 = other acquaintances  6 = people I don't know  7 = therapists  8 = others |
|  | Please specify | enter text | [free text] |
|  | I am online/on the phone with… | Select many | 1 = my partner  2 = my family, my relatives  3 = a friend, some friends  4 = colleagues, classmates, fellow students  5 = other acquaintances  6 = people I don't know  7 = therapists  8 = others |
|  | Please specify | enter text | [free text] |
| **Activity** | What am I doing right now? | Select many | 1 = Work/school/study/training  2 = Resting/relaxing  3 = Everyday tasks/ errands/shopping  4 = Inactive leisure activities (reading, surfing the Internet, social media  media, games, television)  5 = Active leisure activities (sports, hiking, games)  6 = Travelling (by foot, car (passenger seat), public transport)  7 = Entertain myself  8 = Self-care/personal hygiene/  9 = Eat/drink  10 = Caring for others/care work  (children, relatives, partner)  11 = Housework  12 = Nothing  13 = Other ______ (open text field) |
| **Pro-environmental behavior** | Doing so, I paid attention to my energy consumption | VAS | Not at all; very (0-100) |
|  | Doing so, I bought sustainable products (e.g. regionally produced) | VAS | Not at all; very (0-100) |
|  | Doing so, I have used sustainable products (e.g. regionally produced) | | Not at all; very (0-100) |
|  | Doing so, I behaved in an environmentally friendly way | VAS | Not at all; very (0-100) |
|  | I used a bicycle or public transport for this trip | VAS | Not at all; very (0-100) |
|  | Doing so, I paid attention to my waste disposal (e.g. recycled) | VAS | Not at all; very (0-100) |
| **location** | Where am I right now? | select one | 1=At home  2=With family/friends  3= In nature  4= Hospital/ward  5=Work/School  6=Public place  7=Transport  8= Anywhere else (+ open text field _______) |
| **Negative event** | Have you experienced one or more negative events since the last query? | Likert | 1 = not at all, 7 = fully and completely |
|  |  |  |  |
|  | What kind of event was that? | enter text (optional) |  |
| **Emotional resilience** | I had a hard time recovering from that | VAS | Not at all; very (0-100) |
| **Positive event** | Have you experienced one or more positive events since the last query? | Likert | 1 = not at all, 7 = fully and completely |
|  |  |  |  |
|  | What kind of event was that? | enter text (optional) |  |
| **Climate event** | Have you experienced one or more events that reminded you of climate change since the last query? | Likert | 1 = not at all, 7 = fully and completely |
|  |  |  |  |
|  | What kind of event was that? | Select many | 1 = something I have experienced (e.g. high temperatures or flooding)  2= something I have seen on social media (e.g. social media, YouTube)  3= something I have seen in conventional media (e.g. news, newspaper, television)  4= an interaction I have had with someone  5= nothing specific  6= something else |
|  | Please specify | enter text | [free text] |
|  |  |  |  |
| **Value-based living** | Since the last query … |  |  |
|  | I have been able to live by values that are important to me. | VAS | Not at all; very (0-100) |
|  | I knew what I wanted to do with my life. | VAS | Not at all; very (0-100) |
|  | my feelings stopped me from doing what was important to me. | VAS | Not at all; very (0-100) |
| **Self-Efficacy** | Right now, I believe that I can achieve the goals that are important to me. | VAS | Not at all; very (0-100) |
|  | Right now, I could do even difficult tasks well. | VAS | Not at all; very (0-100) |
